# Persistence *versus* Reversion of 3TC Resistance in HIV-1 Determine the Rate of Emergence of NVP Resistance

**DOI:** 10.3390/v4081212

**Published:** 2012-08-07

**Authors:** Barbara A. Rath, Richard A. Olshen, Jerry Halpern, Thomas C. Merigan

**Affiliations:** 1 Center for AIDS Research, Division of Infectious Diseases and Geographic Medicine, Stanford University School of Medicine, Stanford, CA 94305, USA; Email: merigan@stanford.edu; 2 Department of Pediatrics, Division of Pneumonology-Immunology, Charité University Medical Center, Berlin 13353, Germany; 3 Division of Biostatistics, Stanford University School of Medicine, Stanford, CA 94305, USA; Email: olshen@stanford.edu (R.A.O.); jerry.halpern@stanford.edu (J.H.)

**Keywords:** HIV-1, lamivudine, nevirapine, adefovir, resistance, selection, serial passage.

## Abstract

When HIV-1 is exposed to lamivudine (3TC) at inhibitory concentrations, resistant variants carrying the reverse transcriptase (RT) substitution M184V emerge rapidly. This substitution confers high-level 3TC resistance and increased RT fidelity. We established a novel *in vitro* system to study the effect of starting nevirapine (NVP) in 3TC-resistant/NNRTI-naïve clinical isolates, and the impact of maintaining *versus* dropping 3TC pressure in this setting. Because M184V mutant HIV-1 seems hypersusceptible to adefovir (ADV), we also tested the effect of ADV pressure on the same isolates. We draw four conclusions from our experiments simulating combination therapy *in vitro*. (1) The presence of low-dose (1 μM) 3TC prevented reversal to wild-type from an M184V mutant background. (2) Adding low-dose 3TC in the presence of NVP delayed the selection of NVP-associated mutations. (3) The presence of ADV, in addition to NVP, led to more rapid reversal to wild-type at position 184 than NVP alone. (4) ADV plus NVP selected for greater numbers of mutations than NVP alone. Inference about the “selection of mutation” is based on two statistical models, one at the viral level, more telling, and the other at the level of predominance of mutation within a population. Multidrug pressure experiments lend understanding to mechanisms of HIV resistance as they bear upon new treatment strategies.

## 1. Introduction

### 1.1. In vitro ‘Combination Therapy’

Several antiretroviral agents have been licensed to treat infections with the human immunodeficiency virus Type 1 (HIV-1). Antiviral drugs that inhibit RT activity in wild-type HIV-1 select rapidly for drug-resistant variants. Current guidelines therefore recommend the use of several antiretroviral agents concomitantly rather than sequentially [1,2,3].

Two classes of drugs are active specifically against the reverse transcriptase enzyme of HIV-1. The nucleotide analogue RT inhibitors (NRTI) compete with the natural substrate and act as chain terminators in the RT catalytic site. The nonnucleoside analogue reverse transcriptase inhibitors (NNRTIs) are noncompetitive inhibitors that bind exclusively to a hydrophobic pocket in HIV type 1 RT [4]. Interactions between NRTI and NNRTI can be complex and difficult to assess experimentally. 

We established a novel *in vitro* system to test the impact of NRTI pressure on the development of resistance to the NNRTI nevirapine (NVP) in highly NRTI-resistant but NNRTI-naïve isolates.

### 1.2. Lamivudine (3TC) Resistance—The M184V Substitution

When HIV-1 is exposed to the NRTI lamivudine (3TC) at inhibitory concentrations, resistant HIV variants carrying the RT substitution M184V (ATGàGTG) emerge rapidly [5,6,7]. The M184V substitution confers the highest level of resistance (up to 1000-fold) for any NRTI that has been described to date [8]. In cell-free RT assays the M184V mutant virus exhibits altered enzymatic properties. RT with the 184-Val substitution is less able to initiate reverse transcription, to incorporate dNTP, to perform chain elongation, and to undergo compensatory mutagenesis [9,10,11,12,13]. Decreased fitness and increased fidelity of 3TC-resistant virus limit the production of randomly mutant forms, many of which are not viable [14,15,16]. In M184V mutant enzyme, the process of polymerization becomes more accurate; fewer viral variants are produced; and adaptation to environmental stimuli is impaired [17,18,19]. 

### 1.3. Maintaining 3TC Pressure When NNRTI Are Introduced

To simulate an environment that promotes strong evolutionary pressure, we performed serial passages in escalating doses of the NNRTI nevirapine ([NVP]). Resistance against NVP develops rapidly *in vitro* and *in vivo* [20].

We hypothesize that if 3TC pressure is withdrawn and at the same time an NNRTI is introduced, then the M184V mutant strains will be at a competitive disadvantage to the more “fit” and “flexible” wild-type variants, which can adapt to the new drug more easily [19,21]. The concentration of 3TC used in our experiments is within the normal range of wild-type IC50. Since 3TC is known to exert selective pressure on M184V, we would expect inhibition of reversal to wild-type at position 184. In this study we address these questions. Can the anticipated 3TC effect be simulated in multidrug pressure experiments? Does 3TC have the expected impact on reversal and mutation rates, even in the context of high-level 3TC resistance? 

### 1.4. Adding Adefovir (ADV)

We studied the 3TC-induced “antimutator phenotype” [22] further in the presence of adefovir (ADV). M184V mutant HIV has been shown to be hypersusceptible to ADV (as well as its successor, tenofovir) *in vitro* [23]. When ADV is added in the absence of 3TC, does ADV select for wild-type virus at position 184? Does the reversal to wild-type virus at position 184 (M184V-reversal) facilitate the development of NVP resistance? 

The studies reported here come against a backdrop of tension between diminishing viral load through the administration of drugs on the one hand, and constraining viral escape of resistant strains on the other. 

**Figure 1:**
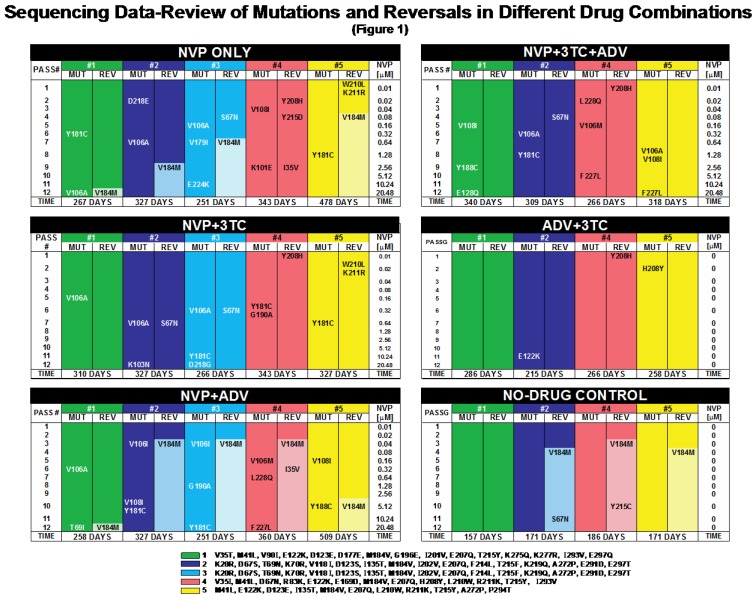
Sequencing Data—Review of Mutations and Reversals in Different Drug Combinations:A complete review of RT sequence changes under the following drug conditions: *NVP_only*, *NVP+3TC*, *NVP+ADV*, *NVP+3TC+ADV*, *3TC+ADV*, *No_drug*. Individual isolates are displayed in different colors: #1 (green), #2 (dark blue), # 3 (light blue), #4 (pink), and #5 (orange). Sequence changes are listed with the passage number (PASS) and [NVP] where they were first observed. Any mutation away from wild-type is listed under ‘MUT’, reversal to wild-type under ‘REV’. Shaded areas within ‘REV’ indicate M184V-reversal.

## 2. Results and Discussion

**Figure 2:**
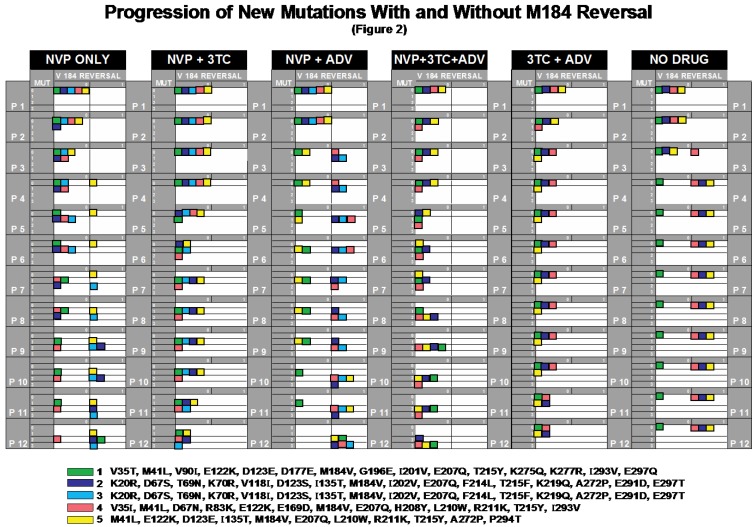
Progression of Mutations With and Without M184V-reversal. All mutations and M184V-reversals for every passage and isolate are summarized. Isolates #1-5 (represented by colored cubes) started with *a priori* no mutations ‘MUT’ and no M184V-reversal (0MUT/0REV). At P1-P12, each cube could either remain in its position or move vertically to 1, 2, or 3 mutations and/or horizontally from 0 to 1 M184V-reversal. ‘Movements’ of cubes under defined drug pressures can be followed along P1-P12 or compared across equivalent passages.

**Figure 3:**
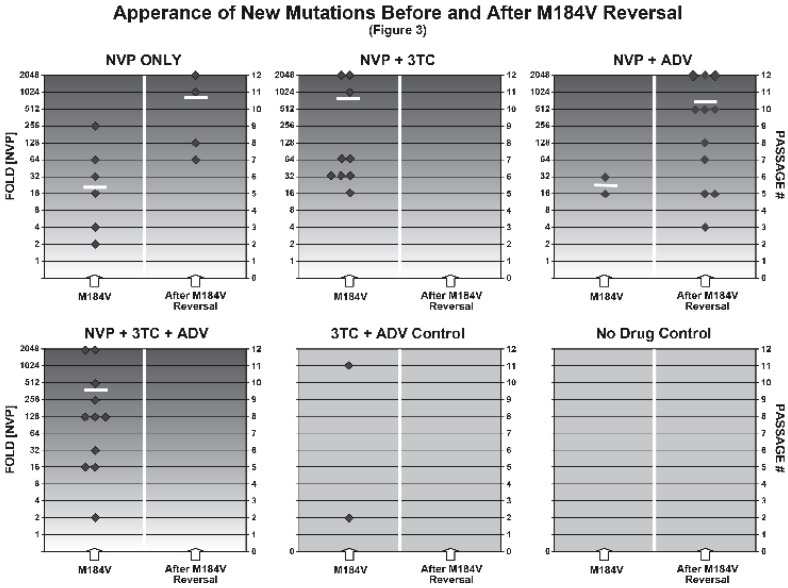
Appearance of new mutations before and after 184 Reversal: All mutations generated (diamonds) per drug setting, before (*M184V*, left column) and after M184V-reversal (*After 184 Reversal*, right column) are summarized. We display the appearance of each selected mutation in relation to the respective passage number and NVP concentration on the y-axis. *FOLD [NVP] *represents the concentration at which a mutation first became prevalent. *FOLD [NVP]* =2^p^ (with P = passage number). As reported in Figure 1, all observed mutations persisted up to P12. During drug escalation, the event of M184V-reversal is required for a mutation to appear in the right column. Diamonds in either column can therefore only be compared between different drug settings. Horizontal bars in the logarithmic *FOLD [NVP] *scale indicate the average [NVP] for mutations to appear.

Figure 1, Figure 2 and Figure 3 summarize explicit baseline mutations and experimental mutations by isolate and drug combination. They also summarize counts of mutations together with incidence of V184M-reversal by isolate and passage number. 

### 2.1. Mutations and Reversals in Different Drug Combinations

Reverse transcriptase (RT) mutational patterns and selected mutations/reversals of the individual isolates #1-5 at baseline and throughout twelve serial passages (P1-P12) are shown in Figure 1, below. All newly selected mutations and reversals persisted throughout P12. No mutations were observed in the *No_drug* control setup. 

#### 2.1.1. Introducing First-time NNRTI in NRTI-Resistant/NNRTI-naïve Clinical Isolates

Baseline isolates #1-5 (see legends) exhibit RT resistance patterns that commonly are observed in salvage therapy, all having changes at positions 184 and 215. It is noteworthy that of 55 baseline mutations, none is known to be in the NNRTI binding pocket, which includes positions 101, 103, 106, 108, 179, 181, 188, 190, 224, 227, and 228. 

All isolates exposed to escalating doses of NVP showed a gradual appearance of one to three mutations, a total of 42 mutations. Of them, 38 were known to be in the NNRTI binding pocket, the remainder are considered noncanonical mutations or polymorphism. All RT mutations were tracked, including those not known to be associated with drug resistance. Please note the remarkably small p-values for the null hypothesis that these 38 mutations were “equidistributed” (that is to say, exchangeable) among possible codons.

#### 2.1.2. Significance of NNRTI Binding Pocket Mutations

For the 19 isolates for which treatment included escalating doses of NVP, there were 38 NNRTI binding pocket mutations. Of these, 13 were at codon 106, seven at 181, and five at 108. The p-values for the findings that the “most popular” codon of 11 had at least 13 mutations, alternatively that the “second most popular” had at least 11, under the common null hypothesis that codons are “equidistributed” (exchangeable) were computed thus.

Isolates are taken to be independent, codons within isolates chosen at random without replacement from among the 11. Reading from isolate #1 through #5, successively from *NVP only* through *NVP+3TC+ADV*, the respective numbers of NNRTI binding pocket mutations were seen to be 2,1,3,2,1,1,2,1,2,1,1,3,2,3,1,2,2,3,3. Therefore, the number of ways NNRTI binding pocket codons could be chosen is


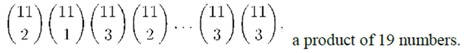


We made this choice at random 50,000 times, each time noting the codon chosen the largest, respectively next largest, number of times, thereby obtaining the joint sampling distribution of these two random quantities. Of the 50,000 trials, the “most popular” codon seen was seen only 12 times, and that occurred for only four trials. The “next most popular” was seen seven times (2056 trials), eight times (129 trials), and nine times (seven trials). It follows that the respective estimated p-values are 1/50,001 (which is about 0.00002) and (2057+129+7)/50,001 (which is about 0.044) when the null hypotheses are as given. The first hypothesis seems untenable, and possibly not the second, either.

#### 2.1.3. Maintaining *versus* Withdrawing 3TC Pressure

Maintaining 3TC Pressure prevented M184V reversal in all instances. Whenever 3TC was withdrawn, we selected for M184V-reversal, except for #4 in *NVP_only* (reversal at 215, 208, and 35 instead) and #1 in *No_drug. *

#### 2.1.4. The Impact of Chance Effects

Isolates #2 and #3 were derived from the same baseline isolate. During lower passage numbers, these two isolates generated similar patterns. The impact of chance effects (‘stochasticity’) on the evolution of these two separate populations became more obvious at later passages. As expected, the two isolates #2 and #3 did not develop identically throughout combination passage experiments, but more similarly than isolates derived from different baseline patient isolates. In all isolates tested, preexisting sequence differences at baseline and viral variants below levels of detection may have contributed to the observed differences in mutational patterns. Since all baseline isolates underwent the same treatments, comparisons can be drawn across treatment groups. 

It is evident that in this experimental setting, selective forces due to increasing evolutionary pressure override the impact of genetic differences at baseline**. **We note that when a mutation was selected at a particular codon for a particular triple, the mutation persisted in subsequent isolates. To be conservative, for “significance” we require a difference in numbers of detected mutations when isolates are compared for a given passage. In the particle model, we also test for whichever treatment has the smaller number of mutations, since the number of particles remains stable with subsequent passage. 

### 2.2. Progression of Mutations with and without M184V Reversal

For improved visualization of HIV evolution and dynamics during serial passage experiments, we summarized *in vitro* responses to different drug combinations in an innovative fashion using a Serial Passage Integrated Display (“Cube Model”) with 2-by-4 tables based on reversal/no reversal and the number of newly selected mutations per clinical isolate (“cube”). 

Figure 2 illustrates how different drug combinations direct the movement of cubes into preferred directions: downward (new mutations), to the right (reversal), or diagonal (both). 

#### 2.2.1. NVP_only

***NVP_only*** simulates the effect of NVP monotherapy and serves as the basic experiment for the comparison with the other NVP escalation experiments. When all isolates in *NVP_only* are viewed together, we see the selection of wild-type at position 184 (M184V-reversal) in most (4/5) cases, though only one through the first six passages. There are 1, 2, or 3 newly selected mutations at least by P12, mostly known NVP resistance mutations (see Figure 1). 

#### 2.2.2. NVP+3TC

With the addition of 3TC (***NVP+3TC***) we observe several changes in comparison to *NVP_only*. M184V-reversal was prevented by the addition of low-level 3TC in all cases despite an exponential increase in NVP doses of up to >2000-fold. In the presence of 3TC, no RT changes could be found when NVP was escalated from P1 through P4 (1 to 8-fold [NVP] and from P7 to P10 (64 to 510-fold NVP). Under extreme pressure (P12, 2048-fold NVP) we observe total numbers of 2x1, 2x2 and 1x3 mutations, but no M184V-reversal. Testing the correlation in the viral particle model, there is a significant difference in the two regimes at the 5% level according to in the suspected direction for isolate #2 at P2 through P5 and for isolate #4 at P4. There are significant differences in the opposite direction for isolate #1 at P5 and isolate #4 at P6. 

#### 2.2.3. NVP+ADV

The addition of ADV to *NVP_only (**NVP+ADV**) *selects rapidly for M184V-reversal (P3). At P12 all isolates have reverted. Moreover, we see a higher total number of NNRTI mutations in comparison to both *NVP_only *and *NVP+3TC*. When *NVP *alone is compared to *NVP+ADV *by the viral particle model, there are significant differences in the anticipated direction for isolate #3 at P3 and for isolate #5 at P5 and P6. There is a significant difference in the opposite direction for isolate #4 at P3. 

#### 2.2.4. NVP+3TC+ADV

***NVP+3TC+ADV ***can be viewed as the 3TC with *ADV+NVP*, as *NVP_only* plus *3TC+ADV, *or as *ADV *with *NVP+3TC; *3TC again prevented M184V-reversal in all cases. However, the addition of ADV to *NVP+3TC* increased the number of NVP mutations selected at lower passage numbers. The high degree of heterogeneity in the presence of ADV was independent of M184V-reversal, which was prevented by 3TC. Comparing *NVP+3TC* with *NVP+3TC+ADV *in the viral particle model, the only significant differences in mutation (other than reversals) are for isolate #4, P2 through P4. They are all in the suspected direction: more mutations appeared when *ADV* was part of the treatment regime. 

#### 2.2.5. 3TC+ADV

***3TC+ADV ***serves as a control experiment; [3TC] and [ADV] were maintained at the same level from P1 through P12. The low number of total mutations suggests that the degree of evolutionary pressure was not comparable with the NVP-escalation experiments; ([ADV] at 2 μM) as an active drug exerted evolutionary pressure and generated mutations. Interestingly, E122K (#2/P11) and H208Y (#5/P3) would not be considered resistance mutations to ADV [24]. 

#### 2.2.6. No_Drug

***No_drug*** simulates a treatment interruption and demonstrates that upon 3TC withdrawal, M184V tends to revert, even if the environment is stable. By P4, 3/4 isolates had reverted. 

#### 2.2.7. Testing the 3TC-Effect

The null hypothesis that 3TC does not lead to altered 184 reversal has p- value 5.982×10-6 , at least if 3TC is specified in advance of the computation. Figure 2 will convince a reader that the attained significance level for any reasonable model should be very small since there was no reversal with 3TC. 

Further inference in this section is devoted to testing the null hypotheses that numbers of mutations result in identical sampling distributions no matter which of two combinations of drugs was administered. In order that tests are conservative, we computed p-values for two-sided alternatives; that is, in principle either combination of drugs could have resulted in a sampling distribution of mutations stochastically smaller or larger than that of the other. 

We employed exact distributions of a Mann-Whitney (equivalently Wilcoxon rank-sum) statistics in computing attained significance [25], respecting that resulting 2×4 tables [with rows representing treatment and columns numbers of mutations (0, 1, 2, or 3)] have many tied observations. Our statistics are, in fact, permutation statistics. When we say “significant” in the table that follows, we mean that the (two-sided) p-value was <0.05. Alert readers will see that we have made no attempt to correct for multiple testing and have not employed false discovery rates [26]. Evidence for our claims is transparent from cursory examination of Figure 1 and Figure 2; we feel that the conservative p-values we supply are sufficient to make our points. (Table 1).

(A) *NVP_only* has significantly more mutations than *3TC+ADV* at passages 8 through 10 and 12.
(B) *NVP+3TC* has significantly fewer mutations than *NVP+3TC+ADV* at passages 9 and 10.
(C) *NVP+3TC* has significantly more mutations than *3TC+ADV* at passages 7 through 10.
(D) *NVP+ADV* has significantly more mutations than *3TC+ADV* at passages 6 through 12.

These are the most extreme comparisons with respective p-values 0 .048, 0.040, 0.032, 0.032, 0.024, 0.032, and 0.008.

(E) *NVP+3TC+ADV* has significantly more mutations than *3TC+ADV* at passages 9 through 12.

**Table 1:** Comparison across passage numbers and drug combinations; testing the null hypotheses that numbers of mutations result in identical sampling distributions no matter which of two combinations of drugs was administered. P-values (<0.05 in **bold**) for comparisons (A) to (E); passage numbers 6–12.

Comparison	Passage Number						
	6	7	8	9	10	11	12
A) *NVP_only versus 3TC+ADV*	0.5238	0.1429	**0.0317**	**0.0476**	**0.0476**	0.0794	**0.0317**
B) *NVP+3TC versus NVP+3TC+ADV*	0.9762	1.000	0.2063	**0.0476**	**0.0397**	0.1190	0.1429
C) *NVP+3TC versus 3TC+ADV*	0.4444	**0.0397**	**0.0397**	**0.0397**	**0.0397**	0.1587	0.0794
D) *NVP+ADV versus 3TC+ADV*	**0.0476**	**0.0397**	**0.0317**	**0.0317**	**0.0238**	**0.0317**	**0.0079**
E) *NVP+3TC+ADV versus 3TC+ADV*	0.3714	0.3714	0.0571	**0.0286**	**0.0286**	**0.0286**	**0.0286**

### 2.3. Appearance of New Mutations before and after 184 Reversal

Figure 3 summarizes the selection of mutations prior to and after M184V-reversal, respectively. Though numerical averages may be of limited descriptive value for random quantities that change by doubling, for completeness these numerical averages are displayed as horizontal white bars. 

***NVP_only ***shows a mixed picture. Mutations were selected at P2, P3, P5, and P6 without prior or simultaneous reversal at position 184 (*M184V*; left column). The first new mutation with reversal (*After M184V Reversal*; right column) became prevalent at P7. More mutations after reversal appeared at P8, P11, and P12, though additional mutations were selected at P7 and P9 without reversal. 

With ***NVP+3TC ***there was no M184V-reversal. With *3TC+NVP* we selected for a total number of 9 mutations, which was the lowest among all NVP escalation experiments. Importantly, the first mutation that appeared with *NVP+3TC* was selected under substantially higher NVP concentrations (P5; 16-fold), by contrast with what was seen with *NVP_only* (P2; 2-fold) or in any other setting. 

With ***NVP+ADV***, mutations appeared after M184V-reversal, the first one at very low NVP concentration (P3; 4-fold). The average [NVP] was lower than in *NVP_only* for both *M184V *(left column, 24-fold *versus* 62-fold) and *After M184V Reversal* (right column: 682-fold *versus* 816-fold). 

In the ***NVP+3TC+ADV ***experiments, 3TC precluded M184V-reversal. The total number of selected mutations (11) was lower than in *NVP+ADV* (13) and higher than in *NVP+3TC* (9). It must be noted, however, that *NVP+3TC+ADV* was done with only four isolates (*versus* five in the other NVP-settings). The first mutation appeared at P2 (2-fold [NVP]). However, the average concentration needed to generate mutations was high (483-fold in *NVP+3TC+ADV*), but lower than without ADV (595-fold in *NVP+3TC*).

### 2.4. Summary of Results

We draw four conclusions from our experiments simulating combination therapy *in vitro*.

1. The presence of low-dose (1μM) 3TC prevented reversal to wild-type from an M184V mutant background.
2. Adding low-dose 3TC in the presence of NVP delayed the selection of NVP-associated mutations.
3. The presence of ADV, in addition to NVP, led to more rapid reversal to wild-type at position 184 than NVP alone.
4. ADV plus NVP selected for greater numbers of mutations than NVP alone.

Inference about the “selection of mutation” is based on two statistical models, one at the viral level, more telling, and the other at the level of predominance of mutation within a population.

### 2.5. Discussion

It is evident from Figure 1, Figure 2 through Figure 3 that during each passage, there is tension between diminishing viral load through the administration of drugs and constraining viral escape through the selection of mutant forms. Our contributions begin with the establishment of an *in vitro* system to study the impact of continued 3TC pressure on the selection of both M184V-reversal and resistance to NVP. 

Several clinical trials demonstrated that the use of genotypic resistance data is associated with improved virologic and clinical outcome in salvage therapy and can be cost-effective [27,28,29]. When sequence data are available to direct the choice of a new regimen and a known resistance mutation is found, it seems plausible that the respective drug has lost antiviral activity and should be discontinued. Specific combinations of antiretroviral agents can exert conflicting genetic pressures [30]. A novel strategy that remains to be established is the continued use of certain individual compounds with the goal to preserve “suppressor mutations” impairing the evolution of resistance to other compounds [4,15,22,31].

Some authors have suggested continuing 3TC therapy even in the context of high-level 3TC resistance [18,19,32,33]. The strategy is to preserve the resistance mutation M184V, which has been linked to an HIV-1 reverse transcriptase with altered biochemical properties. Previous *in vitro* studies have shown that M184V may not delay the emergence of some protease inhibitors (PI) mutations [34], but of some PI and NNRTI [12]. In our study we test for NRTI-NNRTI interactions allowing for structural or functional constraints within the RT enzyme to interfere with the acquisition of new mutations. 

Jonckheere *et al.* compared HIV wild-type to M184V mutant virus with three different stable doses of NVP in the absence of 3TC pressure [35]. Breakthrough of NVP-resistant virus was generally observed one passage later with M184V mutant than with wild-type virus. This study is in agreement with our results, but did not address the effect of concomitant 3TC pressure on diverse clinical isolates. Diallo and Balzarini *et al.* tested the combination 3TC with NNRTI (NVP and efavirenz, respectively) [12,36]. Again, viral breakthrough was delayed significantly when wild-type and 184V recombinant HIV were exposed to 3TC plus NNRTI. The above experiments by Jonckheere, Balzarini and Diallo *et al.* used clonal HIV-1 IIIB laboratory isolates passaged in tumor cell lines, which would be considered the standard method when examining the effect of individual mutations on the emergence of drug resistance mutations. In our experiments we confirmed that this additive 3TC-NNRTI effect is preserved even in the context of “real-life” patient isolates carrying the M184V “naturally”, *i.e.*, after *in vivo* exposure to 3TC. This is even more remarkable as our method used diverse clinical isolates, which may have contained minority variants below the 20% detection level with population-based sequencing [37]. Future developments of this method should employ second generation sequencing methodologies to examine the role of minority variants in the mix, or to compare artificial mixtures of minority variants outcompeting each other under drug pressure.

It remains to be noted that in our assay, we used pooled donor PBMC rather than immortalized laboratory cell lines, aiming to simulate the real-life scenario as closely as possible. While pooled PBMC may pose a potential caveat due to the variability in the composition of PBMC over time, the pooled PBMC culture technique has been developed and established at the Stanford Center for AIDS Research (CFAR) in the early 1990s [38,39,40,41,42] and has since then become part of standard quantitative PBMC culture protocols in the NIAID Virology Manual for HIV Laboratories [43]. Quan *et al.* observed an additive effect of 3TC+NVP in enzymatic assays when measuring the amount of full-length RT product in M184V mutant virus. The authors suggested that 2.5–20 μM 3TC might exhibit a modest antiviral effect in M184V mutant virus despite high-level resistance [32]. Our data are in agreement with this hypothesis, but for 3TC-concentrations as low as 1 μM. 

Our results underline the importance of drug combination testing in patient isolates that are resistant at baseline. In 38 of 42 isolates exposed to NVP we selected for mutations that are located in the NNRTI binding pocket. We note that of them, 13 were at site 106, seven at 181, and five at 108. It seems plausible that structural constraints favor NNRTI mutations in positions 103-108 over changes in positions 181 or 188 as long as M184V predominates. The structure of M184I-HIV-1 has been solved and published [8], NNRTI have been co-crystallized with wild-type as well as Y181C and Y188L mutant RT [44]. For a better understanding of NRTI-NNRTI interactions complex three-dimensional models of dual/triple resistant virus will be required.

In our study we investigate the overall effect of maintaining *versus* withdrawing 3TC pressure in clinical isolates at the first-time use of NNRTI. It is well known that NNRTI mutations are generated quickly *de novo*, even in wild-type laboratory strains. Clinical studies have shown that NNRTI- naïve patients may harbor HIV-1 viral variants with reduced NVP-susceptibility [45,46]. In our study, all isolates were fully susceptible to NVP and ADV at baseline (data not shown). All isolates were exposed to identical experimental conditions, allowing comparisons across the different drugs present in the growth media. In this context it is not surprising that the two isolates (#2 and #3) of common genetic lineage behaved similarly. As we expected, patterns of resistance evolved differently, but always in concordance with one basic hypothesis. That is, no matter the genetic background, 3TC precluded M184V-reversal and impaired the selection of mutations in the NNRTI binding pocket. Thus, continuing 3TC and adding at least two more active agents to the regimen should delay the initial selection of NNRTI resistance. 

The observation that *ADV+NVP* select for greater numbers of RT mutations than *NVP_only* can be explained by the selective pressure of both drugs, *ADV+NVP*, being greater than that of NVP alone, but sufficiently low to allow viral replication and selection. The pressures imposed by ADV would be expected to select changes conferring advantages to replication in the presence of ADV, such as 184 reversion. M184V reversion was promoted by ADV pressure only in the absence of 3TC pressure, contrary to reports of TDV+3TC serial passage experiments in SIV [47]. Interestingly, the mutations observed with ADV in our experiments were, with one exception (T69I), all positioned within the NNRTI binding pocket as opposed to the NRTI binding pocket. Thus, the majority of mutations selected with *NVP+ADV* and *NVP+ADV+3TC* were mutations known to confer NVP, not ADV, resistance. *ADV+3TC* alone selected for random changes at positions 208 and 122. Another NRTI, zidovudine (ZDV) has previously been reported to increase mutation rates [48,49]. 

The rapid outgrowth of 184Met virus in the absence of 3TC in our experiments indicates that fitness disadvantages are compensated for, as soon as drug pressure is released. We screened for replicative fitness in P10-12 supernatants and found that TCID50 values, when measured several times in the absence of drug, were extremely low. Surprisingly, the same isolates showed dramatically improved growth in the presence ADV, NVP and 3TC, independent of the dose range applied (data not shown). Dose-dependent enhancement of viral growth by NNRTI has been reported [44]. The observed phenomenon of dose-independent, but drug-dependent growth enhancement in some of our isolates will be a subject for further investigation [50]. 

It has been suggested that the observed benefits of 3TC in combination therapy, even after 3TC-resistance arises, may be attributed to the net-effect of decreased adaptability and a deficit in viral fitness [5,12,51]. The simulation of combination therapy *in vitro* is a new method that provides an important link between *in vitro* assays and *in vivo* studies in animal models and human subjects. Our data support a chain of evidence derived from biochemical assays and single-drug experiments in laboratory isolates, as we report. We approximate the actual clinical scenario further by using multiple drugs simultaneously in clinical isolates with diverse genetic backgrounds. 

The genetic background has been determined by consensus genotyping as the current method of choice when switching drug regimens. As indicated in the mathematical models used, this includes only the view of majority variants composing >80% of the virus population [37]. In this assay, each virus population was allotted the time required to outgrow drug pressures with each passage before the next passage was started. Future studies using different mathematical models will be addressing growth kinetics during single passages. Allele-specific assays may help determine the role of minority variants in the evolution of drug resistance against combinations of antiviral drugs.

The next level of complexity would be reached in a clinical trial that ought to account for additional parameters, such as patient compliance, virus-host interactions, and the distribution of viral populations within body compartments [31]. Several studies tested the decay of M184V during salvage therapy as well as treatment interruptions [51,52,53,54]. Resistant variants with impaired fitness disappeared within weeks after discontinuation of highly active antiretroviral therapy (HAART), accompanied by rapid viral load rebound. Only well-designed prospective clinical trials can assess the *in vivo* risk/benefit ratio and justify a prolonged, possibly once-daily use of 3TC in 3TC-resistant patients, not only in the context of strategic treatment interruptions, but also when a new regimen is started [31,55,56,57]. 

## 3. Experimental Section

### 3.1. Test Compounds

Lamivudine (3TC) was kindly provided by GlaxoSmithKline (Research Triangle Park, NC), ADV by Gilead (Foster City, CA). NVP was obtained from Boehringer Ingelheim (Ridgefield, CT).

### 3.2. HIV Strains

Six clinical isolates #1 through #5, were cell-free supernatants expanded by cocultivation with donor PBMC (NIAID Virology Manual for HIV Laboratories). They were stored at −70 °C. These frozen stocks were derived anonymously from individuals who had received long-term antiretroviral therapy but who had never been exposed to NNRTIs. The primary samples were sequenced up to RT amino acid position 300. A complete list of initial RT mutations (as compared to the Los Alamos consensus HIV-1B) can be found in the legends of Figure 1 and Figure 2. 

### 3.3. Cells and Cell Culture

Pooled HIV-negative donor PBMC (Stanford Blood Bank) were isolated by centrifugation on Ficoll-Paque and were cultured in RPMI medium containing 15% heat-inactivated fetal calf serum, IL-2, PenStrep and L-Glu. Before use, these cells were stimulated for 2–3 days with phytohemagglutinin (Sigma, St. Luis, MO) and washed [40,41,42].

### 3.4. Passage Experiments

Isolates # 1–5 were set up in five different drug combinations: *NVP_only*, *NVP+3TC*, *NVP+ADV*, *NVP+3TC+ADV*, *3TC+ADV* and *No_drug*, #3 and #2 were aliquots from the same baseline sample, but were run as independent experiments in *NVP_only*, *NVP+3TC*, and *NVP+ADV*. With each passage, the concentration of NVP was doubled. The NVP starting dose was 0.01 mM, around the IC 50 of the NNRTI-naïve baseline isolates. 3TC [1 mM] and ADV [2 mM] were added and maintained at levels around the IC50 of the respective baseline isolates. 

Newly passaged cultures were set up as follows: 100 μl supernatant, incubated with 5 Mio PBMCs in 1ml media without drug. After 2 hours of incubation at 37 °C/5% CO2, the culture was dissolved in 10ml media with the respective drug combinations. The cultures were transferred to 25-mL flasks and again incubated at 37 °C/5% CO2. 

Viral growth was monitored once weekly using a p24 antigen assay on supernatants (Abbott Laboratories, Chicago, IL). At p24 ELISA values <3 × 10^4 ^pg/mL, cultures were split: 2.5 Mio PBMCs were replaced by new donor PBMC in media containing the respective drugs in the same molar concentration. At ≥ 3 × 10^4 ^pg/mL, the cultures were passaged after a 2-hour incubation time. With every passage [NVP] was doubled. The amount of supernatant to infect new cells was adapted according to p24 values obtained before passage. All experiments were carried to the 12th passage (P12), *i.e.*, 2048-fold [NVP], well below cytotoxic levels. Average time to P12 was 293 days (range 157–509 days). With every passage, supernatant was harvested and stored in aliquots at −70 °C. 

### 3.5. ABI Sequencing

Population-based sequencing [37] was done at baseline as well as from the supernatant obtained with every passage. As was described previously [58], purified proviral RNA (Quiagen Viral RNA Extraction Kits, Chatsworth, CA) from cultured cell pellets was amplified by nested PCR. A Superscript-One-Step RT-PCR reagent (Life Technologies, Gaithersburg, MD) was used to obtain DNA segments for sequencing. First-round nested PCR primers were RT-21 [59] and MAW-26 [58] and second-round primers were PRO-1 [60] and RT-20 [59], respectively. Second-round products were sequenced using a dRhodamine labeled terminator kit (PE Applied Biosystems, Warrensburg, UK) and the previously described [61] primers RT-a, RT-b (forward), RT-y and HXBR2-89 (reverse). Sequencing was performed using ABI Model 377 equipment and software. 

Sequences were aligned, proofread, and edited in a blinded fashion. Sequencing data were compared to the corresponding baseline isolates and to the consensus B sequence from the Los Alamos HIV Sequence Database, as well as to data obtained from earlier passages in the same experiment. Any mutation away from consensus B-sequence was defined as mutation. Any mutation back towards consensus HIV-1B was defined as a reversal, even if it was not “all the way back” to consensus. 

### 3.6. Statistical Methods

For a given triple (drug combination, isolate, passage), the individual viral particle is the sampling unit for our first model. Its assumptions are that for each of (approximately) 3x10^4^ viral particles and at each of the 300 RT key codons, the amino acid at that codon is either as it was at baseline or has a mutated value. Based on published estimates, the sensitivity of population based sequencing is at least 80% [37]. We assume that for a fixed codon the probability of mutation is constant across particles [62,63]. For a fixed codon *c*, what might vary for two particles *i* and *i’* is the correlation between the indicator functions of the amino acid values at the codon for the two particles. Recall that the indicator function of an event is 1 if the event occurs and 0 if not. Therefore, the indicator for the *c*^th^ codon of the *i*^th^ particle is 1 if that codon has baseline value. Otherwise it is 0. Symbolically, this is ρ(*i,i’*) = *Corr(I_i_(c),I_i’_(c))*. 

We ask this question: “What would be the maximum value of this correlation, averaged over pairs of particles, so that two isolates differ at the 5% level of significance when one isolate has baseline values at all codons and the other a single mutation away from baseline?” 

The mathematical details and discussion of this novel statistical model are summarized in *3.6.1. Viral Particle Model* and *3.6.2. Population Models*, below:

#### 3.6.1. Viral Particle Model

One approach to testing differences at two fixed passages between two isolates, neither descended from the other, was by what we have termed the *viral particle model*. The basis for comparison at a fixed codon is a two-sample t-like statistic that is the difference of two fractions divided by an estimate of the standard deviation of that difference. We denote the two isolates by *a* and *b*. 

For *a*, say, the indicator of particle *i* having its baseline value at codon *c* is *I_i_(c)*, which has value 1 if *c* is wild type, and otherwise is 0. There is an analogous indicator for *b*. We take the numbers of viral particles to be 30,000. We speak of the correlation *ρ* between two indicators. For two codons *i* and *i′ *we write this correlation *ρ(i,i′).* What matters is actually the value of *ρ(i,i′)* averaged over pairs *(i,i′)* of codons. From the constraint that the variance of a sum cannot be negative, it follows easily that the average *ρ(i,i′)* cannot be less than −3.33 × 10^−5^. In fact we expect that *ρ*, which we cannot know exactly, is positive and small. Because two particles within the same isolate and passage may have replicated inside the same cell, and also because of the physical proximity of any two particles within a flask can vary, we do not assume *a priori* that *ρ* = 0.

For a comparison of differences, the numerator of the t-like statistic is


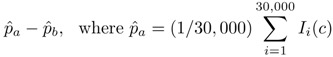


and 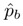 is defined by analogy. 

From a well-known computation with sums of random variables that assume only values 0 and 1 it follows that the variance of 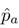, 


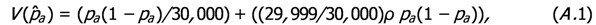


where 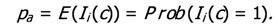


Note that this probability is assumed here not to depend on *i*. (Of course, computations that follow in this appendix, and that are required elsewhere in the paper, show this assumption to be false, decisively. However, the net effect of our assumptions is to make the p-values of our test extremely conservative.) We estimate 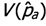 by replacing *p_a_* on the right hand side of (A.1) by 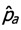.


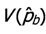
 is estimated analogously. Because 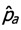 and 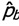 are clearly independent, our t-like statistic is now seen to be


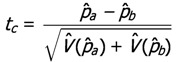


The missing ingredient in *t_c_* is *ρ*, which we admittedly have no way of knowing exactly. But for that, we could approximate p-value for testing the null hypothesis “no difference between given isolates and passages at codon *c*” by *Prob (|Z| > t),* where *Z* is a standard Gaussian random variable; and *t* the observed value of *t_c_*. We could then test the null hypothesis “no difference at any codon” by 300*Prob (|Z| > t*). The latter computation uses the simple Bonferroni bound. In fact what we wish to do with *t_c_* is to find and use the largest value of *ρ* for which the cited 80%–20% difference at some codon for fixed isolates and passages is significant at the 5% level for the null hypothesis as given. First, we solve *Prob (|Z| > t*) = 0.05/300 for *t*, arriving at *t* = 3.7482. 

Then, set 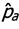 = 0.8, 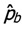 = 0.2, and *t_c_* = 3.7482; and solve for *ρ*. The resulting *ρ* is 0.08.

#### 3.6.2. Population Models

Two further models are based on the notion that for each (drug, passage) combination, isolates are sampling units. Thus, the sample size is five for *NVP_only*, *NVP+3TC*, and *NVP+ADV*, and for *NVP+3TC+ADV*, *3TC+ADV*, and *No_drug*, for a total sample size of 27 for each cited pair. 

Numbers of mutations across isolates within a particular passage for a “counting process” such as ours might be taken to be what is conventional in such applications, a Poisson process. The Poisson model arises when there are many chances for “success” but few “successes”, and in addition trials are independent. These assumptions might apply when we take mutations themselves as sampling units. The presence of mutation or mutations within an isolate and drug combination can be assumed independent across passages; they are certainly independent across isolates. A sum of independent Poisson random variables has a Poisson distribution no matter the respective parameters of the summands. Conversely, if a sum of independent random variables has a Poisson distribution, then according to D. Raikov [64], each summand has a Poisson distribution. Therefore, we can test the null hypothesis that the Poisson model applies to numbers of mutations within a drug combination by looking at Passage 12 to see if the distribution of numbers of mutations across isolates is Poisson. We begin with the usual approach to assessing the Poisson model: via the “Poisson dispersion test” [63]. (In a Poisson model the mean and variance are equal as numbers.) The test statistic is proportional to the ratio of sample variance to sample mean. If the Poisson model for mutations were correct for a fixed treatment (or drug combination), but there was a change by isolate in the Poisson parameter with 184 reversal, then there would be evidence for over-dispersed data and thus evidence against a strict Poisson model. Other aspects of the experiment could lead to over-dispersed or under-dispersed numbers of mutations. (Over[under]-dispersion in a model means that the variance is greater [less] than what the model would constrain it to be.) There are some 0s in the sample variances when isolates are pooled within treatments. The ratio of sample variance to sample mean disregards information in the sample mean when the sample variance is 0. This observation and a Taylor series argument not given here led us to use as a test statistic the difference of sample mean and sample variance rather than the usual ratio. On the null hypothesis that the data are Poisson, the difference should be 0 to within noise. Because there are at most five isolates per drug combination, we could not rely on asymptotic distributions computed under the null hypothesis. Instead, we used the parametric bootstrap distribution [62] of the test statistic under the null hypothesis. This amounts to sampling independently from a Poisson distribution with parameter (mean and variance) the average number of mutations observed at the twelfth passage. The resulting distribution is the reference distribution for the cited difference when the null hypothesis is true. We took 1,000 bootstrap samples per drug combination. This approach enabled us to compute p-values for the null hypothesis separately for alternatives of over-dispersion and under-dispersion relative to the Poisson.

When the number of mutations across isolates within a particular passage is hypothesized to have a Poisson distribution and tested as specified, then the p-value for “over-dispersion” is never less than 0.85. However, for the model with “under-dispersion”, the respective p-values are 0.137 for *NVP only*, 0.147 for *NVP+3TC*, 0.058 for *NVP+ADV, 0*.061 for *NVP+3TC+ADV*, and 0.370 for *3TC+ADV*. There were no mutations but V184M reversal (and two other reversals) for the No drug regimen. Clearly, none of the five p- values is less than 0.05. However, when we combine them by Fisher’s technique [65] of summing minus twice the natural logarithms of the p-values and comparing the sum with a chi-square distribution with 10 degrees of freedom, the overall p-value for the null hypothesis of “under-dispersion” comes to 0.03. For this reason we did not use the Poisson model for numbers of mutations. Instead, our test statistic was nonparametric. Given two candidate drug regimes, it was simply the difference between cumulative numbers of mutations, pooled across isolates. The significance of this difference at each passage was assessed by a permutation test [62].

In summary, the first of the two involves a Poisson assumption that is standard for data like ours but that was discarded after careful study. 

The second of the two models involves a nonparametric statistic. That is, given two candidate drug regimens, it is simply the difference between cumulative numbers of mutations, pooled across isolates. The significance of this difference at each passage was assessed by a permutation test [62]. That the passages of the isolates are independent conditional on their origin is all that matters for the validity of this second test. Sampling distributions of reversal by isolate ought to be closer for the second and third isolates than for any other pair when they are exposed to the same challenges. With the second of the cited two approaches here, we can assign separate p-values for the null hypothesis of “no difference in mutation rate” *versus* each of the two alternatives where one treatment produces a greater mutation rate than the other. 

The issue of whether 3TC precludes V184M reversal can be approached by means of a 2-by-2 table and Fisher’s exact test [63]. One of rows or columns corresponds to “treatment with 3TC or not” and the other to “reversal” or not. There are 27 isolates (Figure 2).

## 4. Conclusions

Using a novel *in vitro* assay and statistical model, we explored useful strategies of combining antiretroviral drugs with potentially divergent effects on the RT substitution M184V, exerting high-level resistance to 3TC.

We noted a pronounced “antimutator effect” when continuing low-dose 3TC while introducing a first-time NNRTI (NVP) in NRTI resistant/NNRTI naïve clinical isolates. Even in the context of high-level resistance, maintaining 3TC pressure prevented reversal M184V in all instances while delaying the emergence of NNRTI resistance. The opposite effect was exerted by ADV; M184V mutant HIV-1 has previously been shown to be hypersusceptible to ADV (as well as its successor, tenofovir) [23].

For improved visualization of HIV evolution and dynamics during serial passage experiments, we summarized *in vitro *responses to different drug combinations in an innovative fashion using a Serial Passage Integrated Display (“Cube Model”, Figure 2) with 2-by-4 tables based on reversal/no reversal and the number of newly selected mutations per clinical isolate (“cube”).
